# Effect of Video Modeling With Simulation on Improving Menstrual Hygiene Skills for Adolescents With Autism Spectrum Disorder

**DOI:** 10.7759/cureus.62847

**Published:** 2024-06-21

**Authors:** M. Arun Kumar, Shweta N.

**Affiliations:** 1 Occupational Therapy, Saveetha College of Occupational Therapy, Saveetha Institute of Medical and Technical Sciences, Saveetha University, Chennai, IND

**Keywords:** simulation, video modeling, pad replacement skills, menstrual practice needs scale, menstrual care skills, pubertal development, menstruation

## Abstract

Background

Menstruation is a biological process experienced by women every month. This project intends to improve menstrual hygiene skills using video modeling with simulation. Educating adolescent girls with autism spectrum disorder to engage in personal care during their menstrual cycle, particularly sanitation and hygiene, is essential. It is important to develop the knowledge and skills necessary for effective self-care during menstruation to prevent sexual health problems. Additionally, the project aims to provide a safe environment for adolescents to practice their menstrual hygiene routines and relieve the stress from their caregivers.

Aim and objective

The study aims to evaluate the effect of video modeling with simulation to improve menstrual hygiene skills in adolescents with autism. The ultimate objective of the study is to determine whether video modeling with simulation in the experimental group enhances the practice of menstrual hygiene among adolescent girls with autism, as well as the impact of traditional occupational therapy intervention on improving menstrual hygiene in this population.

Methodology

This is a quasi-experimental design with convenience sampling and selected samples (n=50), who were then split into two groups: an experimental group (n=25) and a control group (n=25) based on the inclusion and exclusion criteria. The experimental group received video modeling and simulation, while the control group did not receive any specific intervention except parent education and pictorial representations. Pre- and post-tests were conducted to measure the changes. Indian Scale for Assessment of Autism was the screening tool used and the Menstrual Practice Needs Scale (MNPS) was administered. The duration of the study was six months, three sessions per week, lasting 45 minutes to an hour each. The statistical analysis was done with significance at a 1% alpha level using IBM SPSS Statistics for Windows, Version 26 (Released 2019; IBM Corp., Armonk, NY, USA).

Results

The pre-test and post-test data were analyzed using the Wilcoxon signed-rank test and the Mann-Whitney test. The results demonstrated the comparison of the pre-test and post-test mean scores of the MNPS scores were highly statistically significant (p-value of 0.000) when compared to the control group. Following the implementation of video modeling with simulation, the experimental group's post-test scores were significantly higher than the control group's (p-value of 0.000). Thus, the study showed that video modeling with simulation improved menstrual hygiene in adolescents with autism spectrum disorder.

Conclusion

The clinical significance of this study was that the adolescents were excited to watch the videos and perform the activities; furthermore, after video modeling with simulation was implemented, there was a significant improvement in the experimental group when compared to the control group. This enhances the practice of the menstrual hygiene skills independently by the adolescents. Video modeling with simulation has paved the way for improving menstrual hygiene in adolescents with autism. Although the findings from the study are positive, more clinical trials are needed to prove that video modeling with simulation can be used as a therapeutic modality.

## Introduction

Occupational therapy in children promotes their participation in daily life skills to improve their quality of life. A child’s roles include developing independence in their activities of daily living, becoming productive, and participating in play, academics, and leisure skills. The inability to participate because of their disability can cause social isolation, marginalization, and lowered self-esteem [1]. Autism spectrum disorder is a neurological condition characterized by a wide range of symptoms that fall into two groups: restricted behavior, such as difficulty in interacting with others, and stereotypical repetitive functions [2]. It may also coexist with other neuropsychiatric disorders including seizures, Tourette’s syndrome, etc. The behavioral characteristics can be categorized into sub-clusters of disturbances: disturbances in social interaction, communication, behavior, sensory, and perceptual processing [3]. According to the World Population Review, it is estimated that the prevalence of autism spectrum disorder in India is 88.5 in 10,000 children [4].

People with or without autism all experience the physical and mental changes of puberty. However, for female adolescents with autism spectrum disorder, it's particularly challenging as they require more time to cope with the changes that occur in their bodies and often face difficulty with menstrual hygiene management. The need to teach feminine care to girls with developmental delays now occurs at younger ages than ever before [5]. The taboo of intimate hygiene has often caused practitioners to avoid teaching these skills, perhaps leaving them more vulnerable to sexual assault and other medical conditions [6]. Menstrual hygiene, dysmenorrhea, and premenstrual syndrome are common issues in women that could potentially cause the child to discontinue school or take frequent leave, negatively impacting the girl's ability to learn and creating additional stress on her caregivers [7]. In some cases, if heavy bleeding or the cycle's interference with daily activities were the main concern, then non-steroidal anti-inflammatory drugs, hormonal therapy, and oral contraceptive pills were initiated [8,9].

Girls with special needs may take longer to learn the skills required for menstrual management. The assessment of these difficulties includes ensuring the adolescent has the information, support, and opportunity to learn and practice the skills required to be as independent as possible in her self-care. There is extensive literature showing that disabled people face barriers to accessing appropriate water, sanitation, and hygiene services [10]. The social stigma and lack of knowledge about disabilities in our culture mean that families of children with autism spectrum disorder must deal with stress. There is a critical need for comprehensive information for adolescents with autism spectrum disorder, as a lack of such information has been identified as a key factor increasing their vulnerability in safe sexual relationships [11].

Video modeling is one such teaching procedure that can be incorporated into teaching menstrual hygiene skills. It involves an individual viewing a videotaped sample of a model performing a specific activity or task. After viewing the video, an individual is directed to perform the activity observed in the video. A meta-analysis of 23 studies that used video modeling in participants diagnosed with autism spectrum disorders found that both procedures were effective in developing skill acquisition in participants [12]. The menstrual hygiene management packs were developed to promote a gender-friendly school environment and maintenance of intervention facilities. These packs included reusable cloth pads, underwear, carry bags, puberty-related booklets, teacher's training to deliver puberty sessions, and the provision of disposable pads in the school [13].

Simulation also provides support for the development of procedural skills in medical and health professional education. It enables the practice of psychomotor skills in a closed environment. This combination of simulation and teaching/learning methodology has the potential to improve patient care and safety [14]. Psychomotor skills contain components from three learning domains: psychomotor, cognitive, and affective [15]. Despite the ubiquity of the need for these skills, only a few published studies address their importance in this area [16]. Further investigation is needed to understand the use of video modeling to improve menstrual hygiene skills.

It is important to develop the knowledge and skills to provide effective self-care during menstruation to avoid potential sexual harassment and short and long-term sexual health problems. A systematic review [17], covering studies from 2012 to 2017, found that only two studies utilized video modeling to improve the menstrual health of young adolescent girls; hence, it is essential to develop these skills using video modeling in adolescents with autism spectrum disorder. This research gap highlights the importance of the study in this area to support autistic adolescents in performing their menstrual hygiene independently as they age. By addressing these knowledge gaps, researchers can develop more effective interventions and strategies to promote the quality of life for adolescents with autism.

## Materials and methods

**Figure 1:**
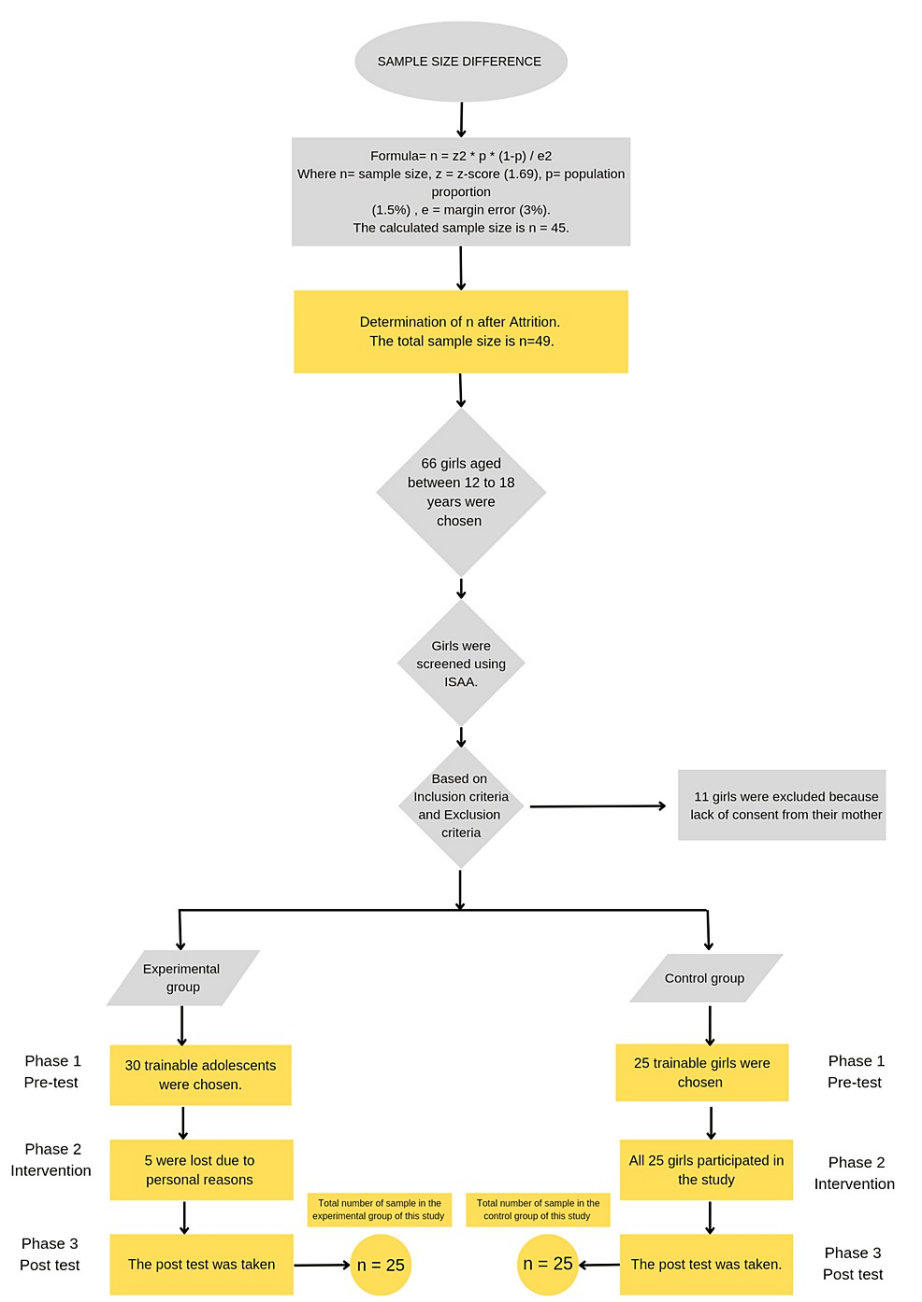
Sample difference ISAA: Indian Scale for Assessment of Autism

The study received ethical approval from the Institutional Scientific Review Board of Saveetha College of Occupational Therapy, Chennai, India (Ethical Clearance No: SCOT/ISRB/005/2023). This research is a quantitative study with a quasi-experimental study design. This quasi-experimental study included an experimental group and a control group. This research was conducted at Prema Vasam and Montford School in Chennai. The sampling technique used was probability convenient sampling. Adolescent girls with mild autism, aged between 12 and 18, were screened using the Indian Scale for Assessment of Autism (ISAA) and chosen. The sampling was based on a predetermined population area. The sample size, n=45, was calculated using the formula n=z^2*p*(1-p)/e^2, where z=1.5% (population proportion) as found in a previous article for a confidence level (α) of 90% [18], and 3% was chosen as the margin of error. The attrition rate for autism spectrum disorder was found to be 7%. Therefore, a total sample size of n=49, in the range of 12 to 18 years, was determined. Initially, 66 adolescent girls were chosen for this study based on the inclusion criteria, which involved adolescent girls with mild autism who had already started their menstrual cycle, adolescent girls whose caregivers, especially mothers, expressed dissatisfaction over the subject's incapability to perform adequate menstrual hygiene practices, and those girls who had not received any medical treatment that could interfere with or suppress the menstrual cycle. None of the screened adolescent girls met the exclusion criteria, which involved excluding adolescent girls from the study who had moderate or severe autism, its comorbid features, adolescent girls who could not even comprehend basic commands, and those who were completely dependent on their caregivers for toileting skills. While getting consent, 11 girls were excluded since their mothers refused to permit any of the interventions, including video modeling with simulation or even conventional occupational therapy. Then the 55 adolescent girls were further divided into 30 girls in the experimental group and 25 girls in the control group. The 25 girls in the control group continued to attend therapy till the end, whereas in the experimental group, out of 30 girls, five girls withdrew due to personal reasons; therefore, the study was completed with 25 girls in the experimental group (Figure 1).

**Figure 2:**
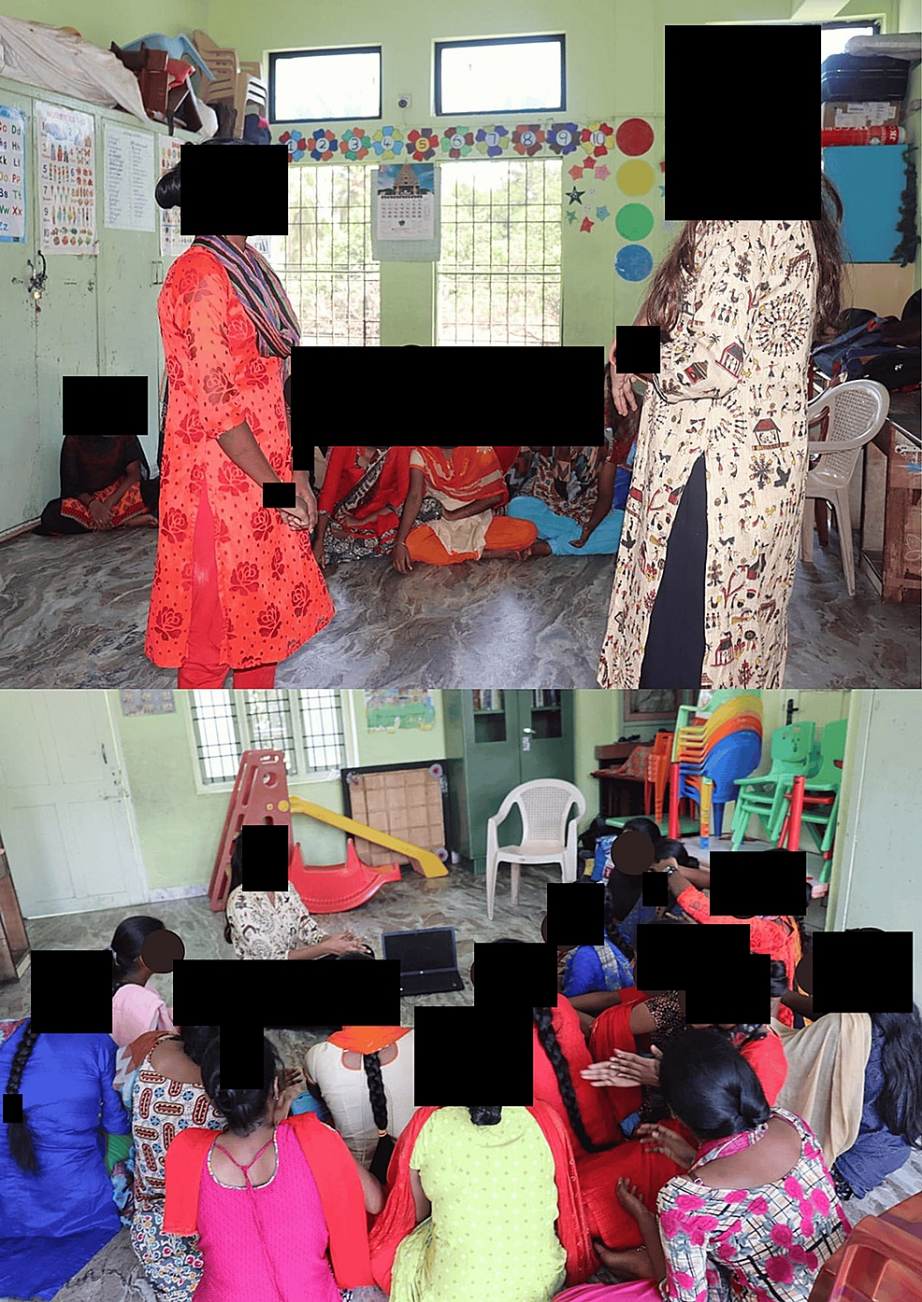
Ice-breaking interaction and warm-up activities

The experimental group was given video modeling followed by menstrual hygiene-based activities and was trained to use disposable pads. The purpose of the study was described to the mothers, and consent to participate in this study was received. Initially, the parents were educated and given an oral presentation about the importance of following menstrual hygiene protocols for their adolescents. The duration of the research was six months, and therapy was planned for three times a week, with a total of 72 sessions. The first two sessions included the pre-test, administered using the Menstrual Practice Needs Scale (MPNS), a comprehensive measure of menstrual self-care that evaluates the ability to perform menstrual hygiene tasks and manage menstruation, including the disposal of used materials. The videos used in this study were designed specifically to improve the process of menstrual hygiene and pad replacement skills. It included an animation video introducing the process of menstruation and then tailored videos explaining each step. The session began with ice-breaking interaction and warm-up activities. These activities helped boost the girls before progressing to the next steps (Figure 2).

**Figure 3:**
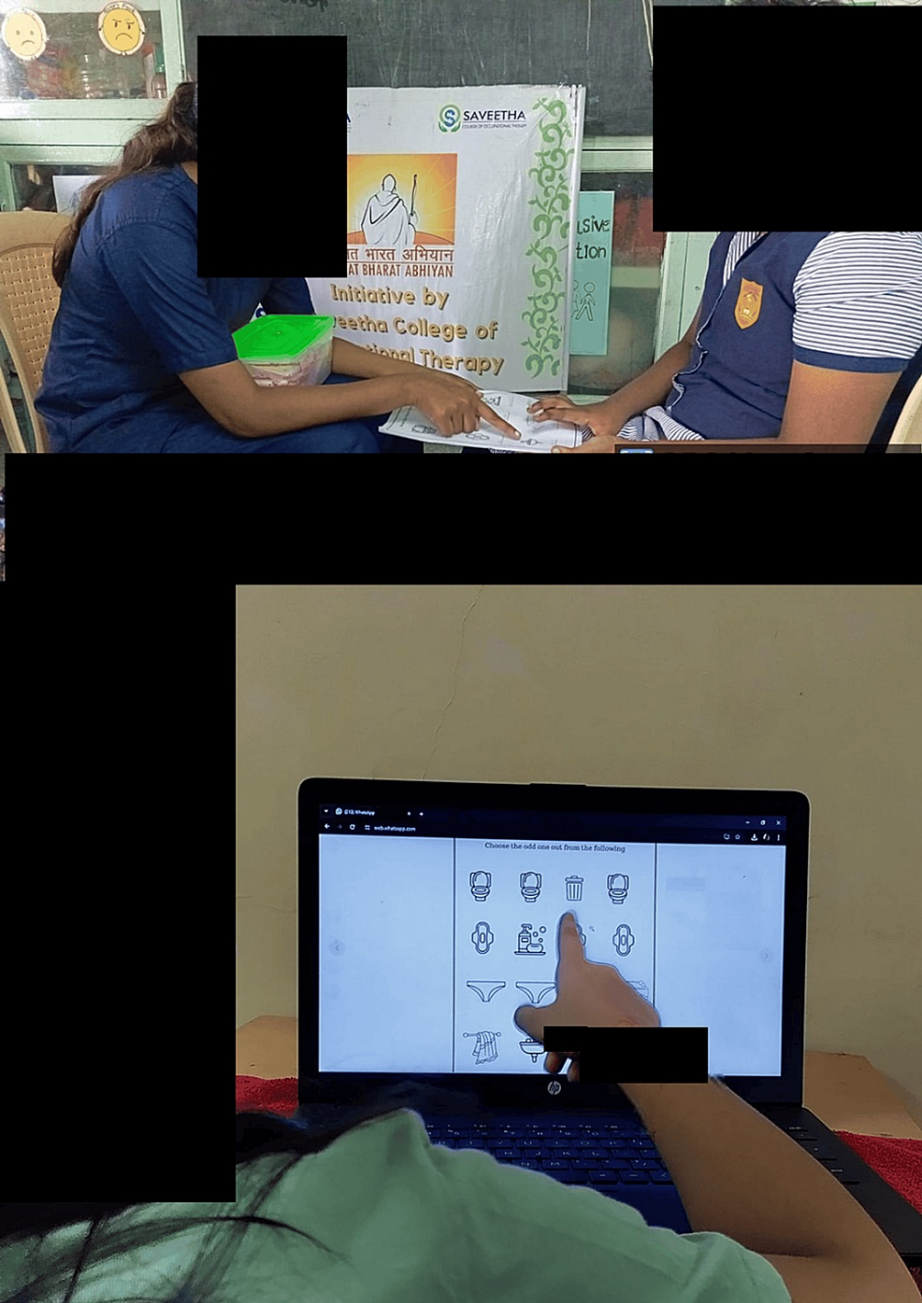
Introduction to the worksheets

Then, followed by worksheets given to the adolescent girls, which focused on the items related to menstrual hygiene, making them understand and learn about the products involved in practicing these skills (Figure 3).

**Figure 4:**
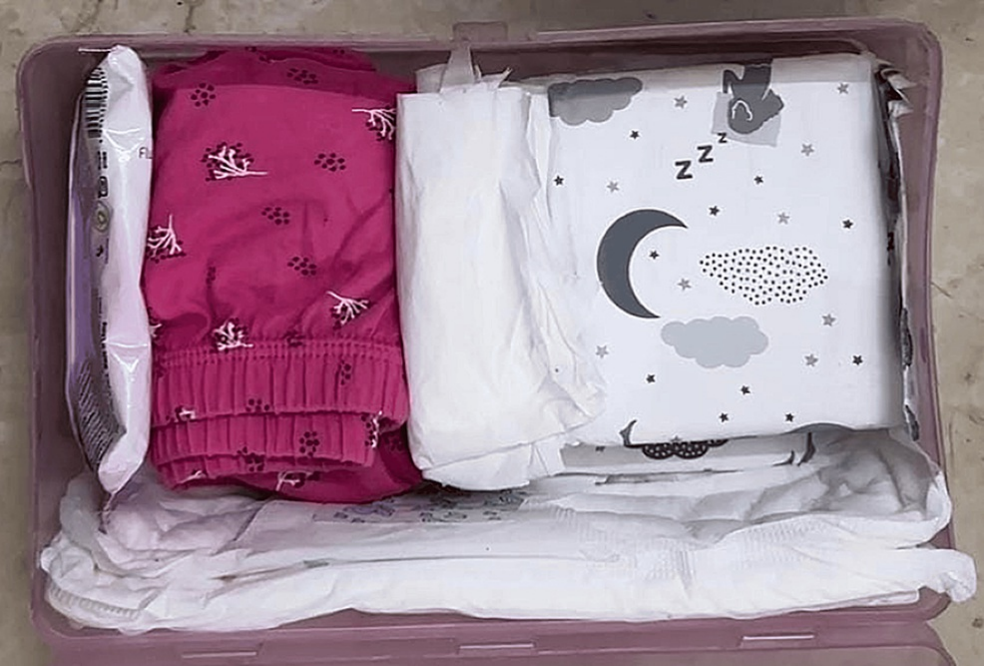
Menstrual hygiene kit

The steps included an introduction to the menstrual hygiene kit. This kit was designed especially for adolescent girls with autism spectrum disorder, where all the products required for executing menstrual hygiene skills were kept together in a box placed in the restroom (Figure 4).

**Figure 5:**
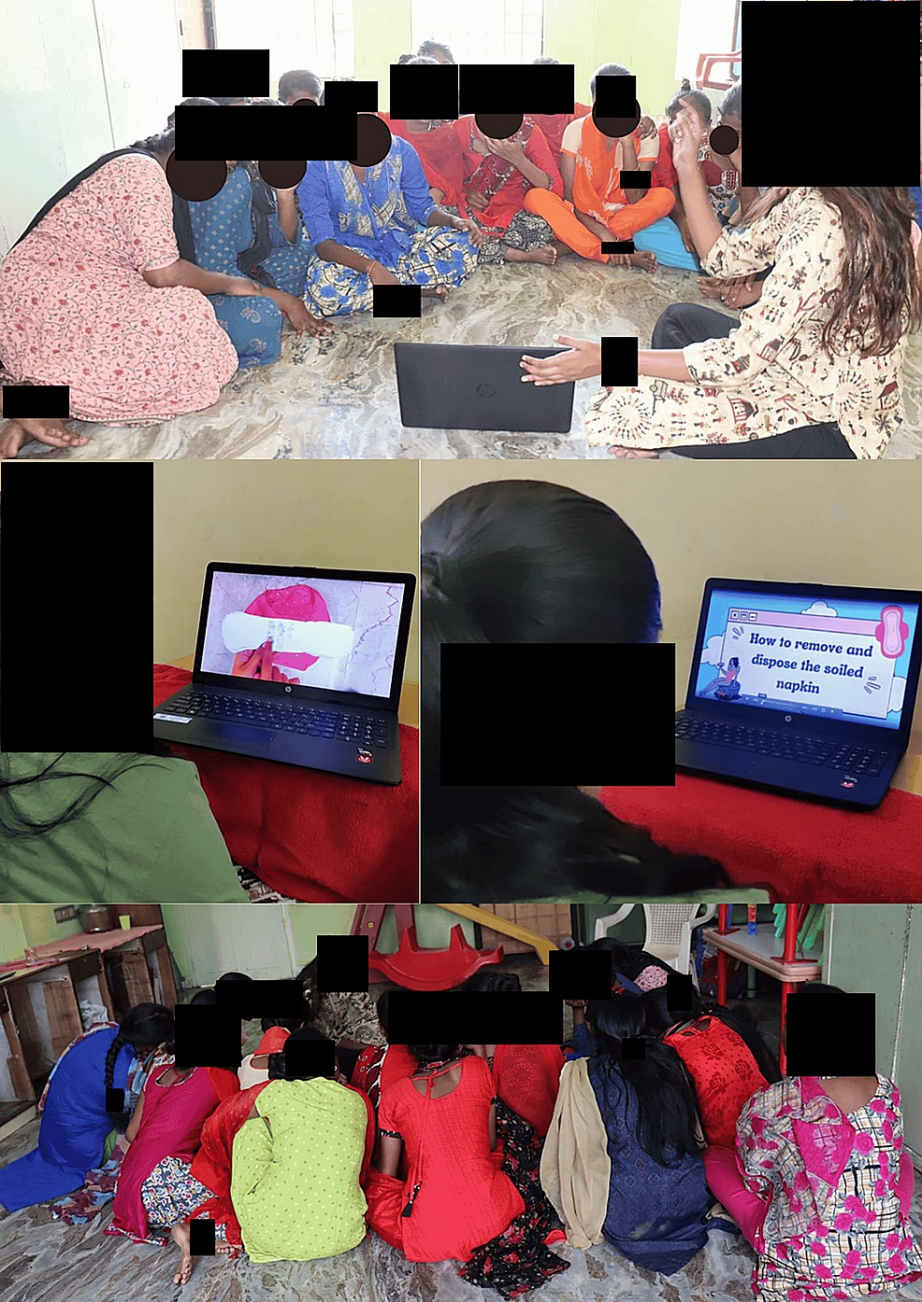
Video modeling

**Figure 6:**
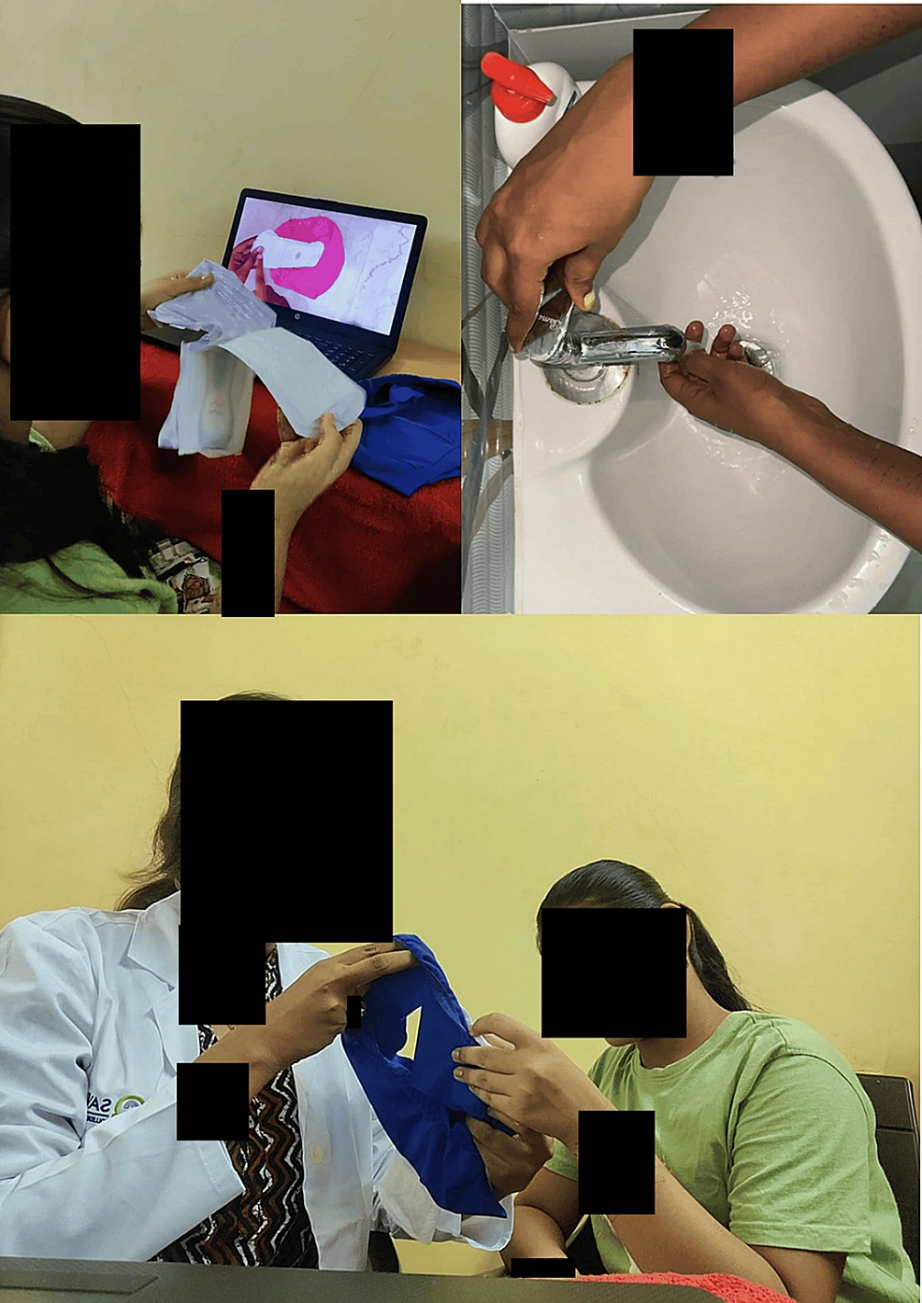
Simulation

Then, choosing the sanitary napkin that is to be stuck on the underwear, unwrapping the sanitary napkin, placing the napkin on the underwear properly, wrapping the soiled napkin in either a newspaper or a disposable cover, disposing of it in the dustbin, and proper handwashing after the procedure. Each video was displayed to the girls, and then the steps were simulated by simultaneously watching the visuals and practicing the steps. The video was played repeatedly until they mastered the skills (Figures 5-6).

The girls were encouraged and received verbal praise after the steps were executed well. Repetition and practice for each skill is mandatory. In the consecutive sessions, the adolescent girls revised the previous steps before moving ahead with the next skill. Simultaneously, the control group underwent health education, parental guidance, and pictorial representations regarding practicing the steps of menstrual hygiene. During the activity, the girls were reinforced, which motivated them to perform better. The parents were also educated about health safety. At the end of six months, the control and experimental groups underwent a post-test, which included the administration of the MPNS scale. The mothers were also informed about the progress of the girls, including their performance initially and their improvement in skills in each session. The mothers were also encouraged to motivate their girls to perform the skills independently. The data collection methods used in this research included a standardized assessment and a questionnaire. Menstrual hygiene was assessed using the MPNS with a reliability of 0.78 and a validity of 0.89. Mild autistic adolescent girls were screened using the ISAA with a reliability of 0.83 and a validity of 0.85. The psychometric properties and the confirmatory factor analysis showed a good fit [19,20]. The statistical analysis was done with significance at a 1% alpha level using IBM SPSS Statistics for Windows, Version 26 (Released 2019; IBM Corp., Armonk, NY, USA) [21]. Both the Mann-Whitney U test and the Wilcoxon signed-rank test were applied.

## Results

Statistical analysis of the control group

In Table 1, since the p-value of 0.007 is less than 0.01, there was a statistically significant difference in the pre-test and post-test scores in the control group of MPNS, indicating an improvement in menstrual hygiene due to conventional occupational therapy intervention.

**Table 1:** Statistical analysis of pre-test and post-test of MPNS in the control group MPNS: Menstrual Practice Needs Scale * Significant at 1% level

		Mean	N	Z-value	p-value
MPNS control group	Pre-test	12.04	25	-2.714	0.007*
	Post-test	12.4	25		

Statistical analysis of the experimental group

In Table 2, since the p-value of 0.000 is less than 0.01, there was a statistically significant difference in the experimental group between the pre-test and post-test scores of the MPNS. Hence, there was an improvement in menstrual hygiene through video modeling with simulation for the experimental group.

**Table 2:** Statistical analysis of pre-test and post-test of MPNS in the experimental group MPNS: Menstrual Practice Needs Scale * Significant at 1% level

		Mean	N	Z-value	p-value
MPNS experimental group	Pre-test	24.76	25	-4.437	0.000*
	Post-test	32.24	25		

Statistical analysis between the control and experimental groups

In Table 3, since the p-value of 0.000 was less than 0.01, there was a statistically significant difference in post-test scores between the experimental and control groups in the total MPNS score. This suggests that the intervention received by the experimental group had more improvement when compared to the control group.

**Table 3:** Statistical analysis between the post-test scores MPNS of the control and experimental groups MPNS: Menstrual Practice Needs Scale * Significant at 1% level

		Mean	N	U-value	p-value
MPNS post-test	Control group	13	25	-6.076	0.000*
	Experimental group	38	25		

## Discussion

The study aimed to determine the effect of video modeling with simulation to improve menstrual hygiene skills in adolescents with autism spectrum disorder. Data from each pre-test and post-test were compared. Furthermore, the significance was established at p<0. An alternate hypothesis was accepted since the p-value of 0.000 is less than 0.01. Hence, there was a statistically significant difference in the experimental group's pre-test and post-test, substantially surpassing the control group in showing results with the improvement of menstrual hygiene, according to the results. This suggested that engaging in video modeling with simulation may be a more effective way to improve menstrual hygiene than any other undergoing traditional health education sessions. These outcomes demonstrated the potential advantages of using video modeling techniques in the intervention meant to enhance menstrual hygiene in adolescents.

Table 1 shows the difference between the pre-test and post-test scores of the control group, which determined a statistically significant enhancement administered by MPNS, with a p-value of 0.007, which was less than 0.01. This suggested that the intervention received by the control group showed improvement, and this result was supported by Haque et al., who investigated implementing educational-based programs where the children learned about the steps of practicing menstrual hygiene, and the use of pictorial representations to demonstrate each skill ideally showed a slight improvement in the menstrual hygiene skills in the children [22]. Additional investigations indicated that girls who received occupational therapy training performed better at menstrual hygiene practices and were able to follow the process precisely, making it accessible to a wide variety of populations in various environments [23,24].

Table 2 shows the result of the statistical analysis of the experimental group's pre-test and post-test scores, which determined a statistically significant enhancement administered by MPNS, with a p-value of 0.000, which was less than 0.01. Previous research implicated the same results, where video modeling, along with some strategies to ensure cleanliness and good hygiene, are important for girls and women to significantly improve menstrual hygiene skills. By implementing this strategy, the girls can independently manage their feminine hygiene routine and reduce the probability of getting genital infections. This, in turn, reduces the stress on the caregivers, thereby reducing their dependency on them [25]. It was suggested that the intervention received by the children in the experimental group also implemented a combination of social story intervention where the steps of executing menstrual skills in a series of stories are combined with visual task analysis to teach menstrual hygiene skills. The objective data provided evidence to support video modeling with simulation to improve menstrual hygiene skills. Further findings also stated a statistically significant increase in the scores of menstrual skills after the training [26,27].

Table 3 shows the difference between the post-test scores of experimental and control groups administered by MPNS, which determined a statistically significant enhancement in post-test scores, with a p-value of 0.000, which was less than 0.01. These findings were supported by a study that stated that there was an increase in the rates of correct performance after the training in a series of steps using video modeling and reinforcement strategies, which are behavior modification techniques that help to improve the motivation of the girls to perform the skills and encourage them after each step [28]. This result was also highlighted by various studies, which evaluated the acceptability and social validity of a caregiver-mediated intervention for youth with autism spectrum disorder for menstrual hygiene management. Utilizing behavior skills training, animated video modeling, and task analysis of breaking it down into simpler steps to improve changing the pad, the study was proven to be a useful and socially valid tool to improve for autistic children. The study claimed that there was a positive impact of menstrual hygiene management workshops on adolescent females with special needs [29,30].

We agree that the small sample size contributes to the limited generalization of our findings. Further research should employ larger and more diverse sample sizes. Some adolescents could not attend the sessions regularly and took breaks in between, which should also be avoided. It is widely understood that adolescents perform differently in comprehending commands while performing the skills of menstrual hygiene. Additionally, a larger sample size would also provide the opportunity to collect multiple data for each construct to employ more complex statistical approaches and enable more accurate evaluation. To support and enhance our findings, future studies should utilize the use of larger sample sizes and a wider range of samples for other neurodevelopmental conditions.

## Conclusions

The most clinically significant finding of the study was that the participants were excited and eager to engage in watching the videos and simulating each step. Furthermore, there was a significant improvement in the experimental group, when compared to the control group in menstrual hygiene skills. This improvement will further improve functions like pad replacement skills, washing hands properly, and proper disposal of the pads after use. Video modeling with simulation appears beneficial for improving menstrual health, hygiene, and quality of life for autistic adolescents. Although the findings from the studies are mostly positive for therapeutic and health benefits, more clinical trials are needed to assess whether video modeling with simulation activities can be used as a therapeutic approach.
